# Infectious Diseases Clinician's Variation in the Management of Pediatric *Staphylococcus aureus* Bacteraemia and Equipoise for Clinical Trials

**DOI:** 10.3389/fped.2019.00249

**Published:** 2019-06-18

**Authors:** Anita J. Campbell, Steven Y. C. Tong, Joshua S. Davis, Alasdair P. S. Munro, Christopher C. Blyth, Asha C. Bowen

**Affiliations:** ^1^Department of Infectious Diseases, Perth Children's Hospital, Perth, WA, Australia; ^2^Wesfarmers Centre for Vaccines and Infectious Diseases, Telethon Kids Institute, Perth, WA, Australia; ^3^Division of Paediatrics, School of Medicine, University of Western Australia, Perth, WA, Australia; ^4^Victorian Infectious Disease Service, The Royal Melbourne Hospital, Peter Doherty Institute for Infection and Immunity, The University of Melbourne, Parkville, VIC, Australia; ^5^Menzies School of Health Research, Darwin, NT, Australia; ^6^John Hunter Hospital, Newcastle, NSW, Australia; ^7^Southhampton Clinical Research Facility, University Hospital Southampton, Southampton, United Kingdom; ^8^Pathwest, QE2 Medical Centre, Nedlands, WA, Australia

**Keywords:** pediatric, *Staphylococcus aureus* bacteraemia, infectious diseases physician, survey, management

## Abstract

**Background:** Pediatric *Staphylococcus aureus* bacteraemia is one of the leading causes of community-acquired blood-stream infection in the developed world; however, our understanding of management practices by treating clinicians is limited.

**Methods:** The authors designed a web-based clinician survey with support from the Australian and New Zealand Pediatric Infectious Diseases group, of the Australasian Society of Infectious Diseases. Clinicians were presented with three pediatric cases of varying severity. Antibiotic choice, durations of intravenous and oral therapy and research priorities for pediatric *S. aureus* bacteraemia trials were gauged.

**Results and Conclusion:** Large variation in antibiotic prescribing amongst clinicians is demonstrated and increased, corresponding with escalating case complexity and persisting MRSA bacteraemia. Most clinicians chose defining optimal duration of therapy for *S. aureus* bacteraemia as their top clinical trial priority. These findings highlight the importance of prioritizing pediatric *S. aureus* bacteraemia clinical trials, to inform guidelines and best practice management.

## Introduction

*Staphylococcus aureus* bacteraemia (SAB) is amongst the most common infectious reason for admission to the pediatric intensive care unit and one of the leading causes of community-acquired bacteraemia in the post-vaccine era (1). Despite this, there remains a void of clinical trial evidence directing optimal antibiotic duration and treatment for pediatric SAB. This clinician-based survey sought to ascertain the spectrum of clinical practice for the management of pediatric SAB, assessing trends in antibiotic prescribing amongst Infectious Diseases (ID) clinicians and exploring key priorities to direct future pediatric SAB trials.

## Materials and Methods

The authors designed and piloted a web-based survey (SurveyMonkey, Sydney, Australia), with support from the Australian and New Zealand Pediatric Infectious Diseases (ANZPID) group, of the Australasian Society of Infectious Diseases (ASID). Respondents were recruited through the ASID email forum from August to September 2016, and January to March 2017.

Clinicians were presented with three pediatric cases, aged <16 years, of varying severity (Supplementary File). Classification of cases were derived from established definitions for adult SAB (2), and published pediatric risk factors for poor outcome (1). Multiple answers to questions and alternative free-text options were permitted where appropriate, including for antibiotic treatment options. Answers were presented in an electronically randomized order. Antibiotic choice, durations of intravenous (IV) and oral therapy and research priorities for pediatric SAB trials were gauged.

## Results

Forty-eight respondents from 100 ANZPID members participated. Fourteen (29%) had been practicing medicine for >20 years and 38 (79%) were qualified ID or Microbiology consultants. Respondents from all pediatric tertiary hospitals across Australia and New Zealand were included.

### Case One: Simple MSSA-Bacteraemia in a Toddler With Septic Arthritis (*n* = 46 Respondents)

Most clinicians selected flucloxacillin monotherapy (27/46, 59%) for empirical management of uncomplicated septic arthritis (Table 1). Empirical MRSA cover was recommended by 41% (19/46), with nine (47%) respondents using local MRSA rates, presence of bacteraemia and previous colonization with MRSA to inform this decision. All preferred IV flucloxacillin for confirmed MSSA-bacteraemia (MSSA-b); for 3 days (7/46, 17%), 7 days (25/46, 54%), or 14 days (9/46, 20%). The total duration (IV and oral) varied from 10 days (1/46, 2%) to 6 weeks (4/46, 9%), with 3 to 4 weeks (31/46, 67%) most commonly selected. Clinicians favored oral step-down with cephalexin (37/46, 80%) over flucloxacillin (9/46, 19%), citing palatability and ease of dosing.

**Table 1:** Principal antibiotic preferences for clinicians surveyed for the management of three cases of paediatric *S. aureus* bacteraemia.

**Case**	**Empirical IV Abx therapy**	**Targeted IV Abx therapy**	**Oral step-down Abx**	**Total duration Abx (IV plus oral)**
1. Simple MSSA-B in a toddler with septic arthritis	Flucloxacillin (27/46, **59%**)	Flucloxacillin (46/46, **100%**)	Cephalexin (37/46, **80%**)	3–4 weeks (31/46, **67%**) Range 10 days to 6 weeks
2. Complex MRSA-B in a 9-year-old with localized infection of bone and adjacent muscle	Flucloxacillin with vancomycin (16/35, **46%**)	Vancomycin with clindamycin (15/35, **43%**)	Clindamycin (24/33, **73%**)	6–8 weeks (19/33, **58%**) Range 4–28 weeks
3. Complex, persistent MRSA-B in an adolescent with multifocal disease	Vancomycin with flucloxacillin (18/38, **47%**)	Vancomycin with clindamycin (18/38, **47%**)	Clindamycin (10/29, **34%**)	12–16 weeks (11/23, **48%**) Range 6–30 weeks

*IV, intravenous; Abx, antibiotic; MSSA-B, methicillin susceptible Staphylococcus aureus bacteraemia; MRSA-B methicillin resistant S. aureus bacteraemia; n, number. Bold values represent the proportion (percent) of clinicians who chose this antibiotic preference for which was the majority*.

### Case Two: Complex MRSA-Bacteraemia in a 9-Year-Old With Localized Infection of Bone and Adjacent Muscle (*n* = 35 Respondents)

Empirical combination MRSA/MSSA treatment was preferred (25/35, 71%), mostly with flucloxacillin/vancomycin (16/35, 46%) or flucloxacillin/clindamycin (9/35, 26%). Directed therapy included combination vancomycin/clindamycin (15/35, 43%), or clindamycin (9/35, 26%) or vancomycin (5/35, 14%) monotherapy. Total duration for MRSA-bacteraemia (MRSA-B) with complicated femoral osteomyelitis ranged from 4 to 28 weeks, with 6–8 weeks favored (19/33, 58%). A 2-week IV component (21/33, 64%) was the leading choice for most respondents, with wide variation from 1 (4/33, 12%) to 6 weeks (3/33, 9%). Preferred oral step-down regimens included clindamycin (24/33, 73%) or trimethoprim/sulfamethoxazole (4/33, 12%). In addition, 40% (14/35) indicated they would consider an adjunctive protein synthesis inhibitor (e.g., clindamycin), when toxin mediated *S. aureus* infection was suspected.

### Case Three: Complex, Persistent MRSA-B in an Adolescent With Multifocal Disease (*n* = 38 Respondents)

In this severe case of *S. aureus* sepsis, all respondents preferred combination empirical MRSA/MSSA therapy with vancomycin/flucloxacillin (18/38, 47%) or flucloxacillin/vancomycin/clindamycin (14/38, 37%). When confirmed as non-multiresistant MRSA-B, most continued combination antibiotics for directed therapy (22/38, 58%), using vancomycin-containing regimens (29/38, 76%), predominantly with vancomycin/clindamycin (18/38, 47%). Persisting MRSA-B with an unchanged vancomycin minimum inhibitory concentration (MIC) of 1.0 mg/L, influenced clinicians to adjust therapy (22/38, 58%) and ten (27%) to move away from vancomycin therapy. When the scenario was altered to include a rising vancomycin MIC of 2.0 mg/L, a further 19 (50%) respondents removed vancomycin from the regimen and six (6/38, 16%) added another antibiotic to an existing regimen. Newer antibiotic agents were favored including; linezolid (20/38, 53%), daptomycin (10/38, 26%) and ceftaroline (4/38, 11%). With persisting MRSA-B and increasing case complexity, the number of different antibiotic combinations chosen increased from 8 to 19, with the majority electing for combination directed antibiotic therapy (34/38, 89%).

Total duration of treatment ranged from 6 to 30 weeks, with 12 (4/23, 17%) to 16 (7/23, 30%) weeks preferred. Most clinicians opted for longer IV therapy for multifocal MRSA-B with iliac vein thrombosis, including four (10/38, 26%) to six (20/38, 53%) weeks.

Two thirds (29/38, 76.32%) chose oral antibiotic step-down in this case favoring: clindamycin (10/29, 34%); trimethoprim/sulfamethozaxole (5/29, 17%) or rifampicin and fusidic acid (4/29, 14%).

For cases two and three, clinicians cited down trending C-reactive protein (CRP) (27/29, 93%), clinical improvement (26/29, 90%) and fever resolution (23/29, 79%) as factors influencing timing of IV to oral switch and total duration of therapy. Conversely factors influencing longer treatment duration included persistent bacteraemia (34/38, 90%), multifocal disease (31/38, 82%), anticipated poor adherence to oral antibiotics (24/38, 63%), persistent fevers (16/38, 42%), an endovascular focus (4/38, 11%), and an undrained focus of infection (3/38, 8%).

### Research Prioritization for SAB Clinical Trials in Children (*n* = 38 Respondents)

Responding by both free-text (26/38, 68%) and ranking answer (22/38, 58%) style questions, most clinicians chose defining optimal duration of therapy for SAB as their top clinical trial priority, followed by antibiotic choice for the treatment of SAB (20/38, 53%), MRSA-B (10/38, 26%), and defining the role of adjunctive therapy for severe *S. aureus* disease (21/38, 58%) (Table 2). Willingness to enrole patients in a trial randomizing to an adjunctive protein synthesis inhibitor vs. placebo, combined with empiric antibiotic treatment for severe SAB had clinician equipoise. Three quarters of respondents were in favor (26/34, 76%), whilst 15% were hesitant (5/34) or unwilling (3/34, 9%).

**Table 2:** Clinician-ranked clinical trial priorities for pediatric *S. aureus* bacteraemia.

**Rank**	**Topic**
1	Optimal duration of SAB antibiotic treatment
2	Defining the role of adjunctive antibiotic therapy
3	Optimal antibiotic choice for SAB treatment
4	Optimal antibiotic choice for MRSA-B treatment
5	Optimal oral antibiotic step-down therapy for *S. aureus* osteomyelitis

*1, highest ranked priority; 5, lowest ranked priority; SAB, S. aureus bacteraemia; MRSA-B, Methicllin resistance S. aureus bacteraemia*.

## Discussion

Treatment decisions by ID physicians managing pediatric SAB showed significant variation, highlighting the paucity of evidence and the importance of developing prioritized research agendas for SAB trials. Most respondents selected anti-staphylococcal penicillins (ASP) for MSSA-B directed therapy, despite observational data supporting cefazolin as non-inferior to ASP (3). In contrast, North American PID physicians report equal cefazolin or ASP use for pediatric SAB (4). The national antibiotic handbook recommendations and a lack of randomized controlled trial (RCT) data comparing ASP with cefazolin support this preference (3).

MRSA-B directed therapy with a vancomycin-containing regimen was selected by only 76% (case three) and 57% (case two) of respondents, despite the Infectious Diseases Society of America (IDSA) guidelines preferring vancomycin as first line therapy (2). This may reflect clinician concern regarding poorly defined pediatric vancomycin dosing targets (2), observational data showing vancomycin therapy as a risk factor for poor outcome in children irrespective of methicillin susceptibility (2), and greater availability of newer alternative MRSA agents.

Linezolid was the preferred alternative for MRSA-B in cases two and three, despite its bacteriostatic status and the IDSA MRSA guidelines cautioning against its use in this setting (2). In support of this preference, salvage therapy studies suggest this distinction *in vitro*, may not correlate with clinical outcome (5). Similarly, observational data supports linezolid's role for the treatment of pediatric skeletal infection (6). Daptomycin and ceftaroline were other frequently selected alternative MRSA agents, in contrast to North American PID physicians who favored daptomycin first, consistent with IDSA guideline recommendations (2, 4). Whilst a RCT of daptomycin vs. standard therapy in children with osteomyelitis is ongoing (www.clinicaltrials.gov NCT01922011), trial evidence in children is limited to the treatment of complicated skin and soft tissue infection (daptomycin and ceftaroline) (7, 8), and the treatment of community acquired pneumonia (ceftaroline) (9).

Optimal duration of therapy for pediatric SAB is poorly defined. This is reflected in the variation in duration of treatment selected amongst clinicians, particularly for persisting multifocal MRSA-B. A recent systematic review found level D (weak), IV (case series/cohort study) evidence for pediatric SAB-treatment duration (3). This review recommended 7–14 days IV duration, with consideration of shorter IV duration (4–7 days) for uncomplicated pediatric MSSA-B with skeletal infection (3). Despite this, 30% of clinicians exceeded this minimum duration for uncomplicated MSSA-B septic arthritis. The randomized trial supporting short course therapy in 131 Finnish children with skeletal infection had, however, an absence of MRSA and excellent outcomes despite minimal surgical procedures (10), which are inconsistent with local clinical experience. This underscores the need for further multi-site trials to evaluate the duration of treatment of pediatric SAB that are inclusive of children with MRSA and severe disease.

Dual empirical MRSA/MSSA treatment data for pediatric SAB are limited. Local resistance rates were cited in decision making by respondents but consensus on the threshold for introducing dual cover varies. Recent European pediatric skeletal infection guidelines (6) recommended empirical MRSA cover where local MRSA rates exceed 10–15%. With local MRSA rates approximating 20% (1), respondents favoring combination empirical therapy varied from 41% (case 1) to 100% (case 3). Other variables including case severity and ethnicity likely influenced decision making.

Some limitations include that surveyed responses may not reflect actual treatment decisions; and complete responses from all surveyed participants were not captured. Study bias was reduced with free text and multiple answer options available where appropriate and randomization of the order of answers presented.

## Conclusion

Large variation in antibiotic prescribing for pediatric SAB is demonstrated amongst clinicians within the same jurisdiction and internationally. In addition, variation in practice increased, corresponding with escalating case complexity and highlights the paucity of evidence directing management. Furthermore, clinician-ranked trial priorities and clinician equipoise summarized from this survey should guide the design of pediatric SAB clinical trials to optimize patient outcomes.

## Ethics Statement

The study was exempt from the above requirement given the survey did not involve patients or their families and was a clinician based survey. Data collected was all non-identifiable information that was presented in a summarized format.

## Author Contributions

All of the authors have contributed to editing the manuscript. AC and AB have contributed to also devising the survey, distributing it, and analyzing the results.

### Conflict of Interest Statement

The authors declare that the research was conducted in the absence of any commercial or financial relationships that could be construed as a potential conflict of interest.
