# Supplementary material for: Infectious Diseases Clinician's Variation in the Management of Pediatric Staphylococcus aureus Bacteraemia and Equipoise for Clinical Trials
Source: Front Pediatr. 2019 Jun 18;7:249. doi: 10.3389/fped.2019.00249 (PMC6611400; doi:10.3389/fped.2019.00249)
Supplement: Supplementary file 1 [file Table_1.DOCX]

**Supplementary File**

**Here are the questions asked, unchanged, from** an online survey, hosted on SurveyMonkey, publicised through Ozbug and ANZPID (Infectious Diseases and Microbiologists email discussion forum) and supported by **the Australasian Society of Infectious Diseases Clinical Research Network (ASID CRN). The survey was open from August to September 2016, and January to March 2017.**

#### **Survey title: Paediatric *Staphylococcus aureus* Management Survey (SAMS)**

#### **Thank you for participating in our survey. Your feedback is important. You will be asked to answer questions on treatment regimens for paediatric**S. aureus**infection based on 3 case scenarios, and some general treatment questions. It will take approximately 15 minutes to complete. No personal/identifying details will be collected. The responses will be used to prioritise future studies in**S. aureus**infections in children in Australia and New Zealand.**

#### How many years of medical practice have you completed? (Overall)

#### **Answers: 0-9yr, 10-19yr, 20-29yr, 30-39yr, 40+ years**

#### Are you a:

#### Answers: RMO/registrar, ID trainee, Micro trainee, ID consultant, Micro consultant, other (please specify)

#### Do you work predominately in paediatric or adult ID/micro?

#### Answers: Paediatric, adult, other (please specify)

#### Answers: w

#### **What is you jurisdiction?**

**Answers: NSW, QLD, Victoria, Tasmania, Western Australia, Northern Territory, Canberra, New Zealand, other** (please specify)

**Section one**

**Case one: *A 2 year old Caucasian boy presents febrile with a swollen, painful, warm, right knee. Blood cultures (BC) collected and joint aspirated and washed out showing > 50, 000 White cells (WCC) and gram positive cocci (GPC) in clusters on gram stain. No evidence of osteomyelitis on x-ray or bone scan.***

#### Your preferred ****EMPIRIC intravenous treatment regimen**** for suspected *S. aureus* disease is? (select ALL antibiotics you would prescribe in your empiric regimen for this patient)

#### Answers: flucloxacillin, cefazolin/cephalothin, vancomycin, clindamycin/lincomycin, cotrimoxazole, linezolid, rifampicin, ceftaroline, daptomycin, gentamicin, other

#### *BC flags with GPC in clusters on Day 1 of admission, subsequently identified as Methicillin susceptible*S. aureus*(MSSA).*

#### Your preferred ****DIRECTED regimen for intravenous**** therapy for septic arthritis and MSSA bacteraemia is:

#### Answers: flucloxacillin, cefazolin/cephalothin, vancomycin, clindamycin (lincomycin), flucloxacillin and vancomycin, flucloxacillin and clindamycin (lincomycin), other (please specify)

#### *Assume an otherwise uncomplicated clinical course in a 2 year old with septic arthritis and S. aureus bacteraemia.*

#### Your preferred ****total duration of intravenous therapy**** for septic arthritis and S aureus bacteraemia is:

Answers: 3 days, 7 days, 10 days, 14 days, other (please specify)

1. Your preferred regimen for oral therapy for septic arthritis and S. aureus bacteraemia in a 2 year old is:

Answers: flucloxacillin, cephalexin, cotrimoxazole, amoxicillin/clavulanic acid, clindamycin, other (please specify)

#### Your preferred total duration of****oral therapy****(not inclusive of the IV component) for septic arthritis and S. aureus bacteraemia in a 2 year old is:

Answers: 1 week, 2 weeks, 3 weeks, 4 weeks, 5 weeks, 6 weeks, other (please specify)

1. Any additional comments about the management of this case that you would like to make?

Answers: free text

**Section two**

Case two: ***A 12 year old Pacific Islander girl presents with fever, swollen, tender right thigh and inability to weight bear. MRI shows femoral osteomyelitis with large subperiosteal collection and associated pyomyositis of the quadriceps muscle.***

#### Your preferred EMPIRIC antibiotic regimen includes: (select ALL antibiotics you would prescribe in your empiric regimen for this patient)

#### Answers: flucloxacillin, cefazolin/cephalohin, vancomycin, clindamycin/lincomycin, cotrimaxazole, linezolid, rifampicin, ceftaroline, daptomycin, gentamicin, other (please specify)

#### *BC flag positive with GPC in clusters on Day 1, subsequently identified as community associated/ non-multiply resistant MRSA. Vancomycin MIC is 1.0mg/L. Susceptible to all antibiotics other than methicillin. The lab confirms that this is PVL+ve. Subperiosteal and pyomyositic collections are drained / washed out on four occasions. No further blood cultures are positive.*

#### Your preferred DIRECTED antibiotic regimen is: (select ALL antibiotics you would prescribe in your directed regimen for this patient)

#### Answers: Flucloxacillin, cefazolin/cephalohin, vancomycin, clindamycin (or lincomycin), cotrimaxazole, linezolid, rifampicin, ceftaroline, daptomycin, gentamicin, ceftobiprole, other (please specify)

1. Your preferred **total duration of intravenous therapy** for this patient is:

#### Answers: 2 weeks, 4 weeks, 6 weeks, 8 weeks, 10 weeks, 12 weeks, other (please specify)

#### Your preferred oral regimen for step down therapy of severe osteomyelitis is (select all that apply):

#### Answers: flucloxacillin, cephalexin, clindamycin, linezolid, cotrimaxazole, fusidic acid, rifampicin, ciprofloxacin, doxycycline, erythromycin, other (please specify)

#### Your preferred total duration of oral therapy (not inclusive of the intravenous component) for this patient is

#### Answers: 2 weeks, 1 month, 6 weeks, 3 months, 4 months, 5 months, 6 months, other (please specify)

**Section three**

Case three: ***A 9 year old Indigenous boy presents with 5 days of fever, cough and swollen, painful left hip. CXR shows evidence of multiple pulmonary abscesses consistent with septic emboli. Left lower limb ultrasound shows evidence of thrombus in the iliac vein. The patient was admitted to ICU with hypoxia requiring intubation and inotropes. They proceed to washout of left hip showing > 50 000 WCC and GPC in clusters on gram stain.***

#### Your preferred ****EMPIRIC intravenous antibiotic**** regimen is: (select ALL antibiotics you would prescribe in your empiric regimen for this patient)

Answers: flucloxacillin, cefazolin/cephalothin, vancomycin, clindamycin/lincomycin, cotrimoxazole, linezolid, rifampicin, ceftaroline, daptomycin, gentamicin, other (please specify)

*BC flag positive with GPC in clusters on Day 1, subsequently identified as community acquired / non-multiply resistant MRSA. Vancomycin MIC is 1.0mg/L. Susceptible to all antibiotics (including clindamycin) other than methicillin.*

1. Your preferred **DIRECTED intravenous antibiotic** regimen (select ALL antibiotics you would prescribe in your directed regimen for this patient)

Answers: flucloxacillin, cefazolin/cephalothin, vancomycin, clindamycin/lincomycin, cotrimoxazole, linezolid, rifampicin, ceftaroline, daptomycin, gentamicin, other (please specify)

***Scenario A:****By day 8, blood cultures remain positive for*S. aureus*with vancomycin MIC unchanged at 1.0mg/L. Patient remains intubated and ventilated in ICU. Transthoracic echo does not show evidence of endocarditis. Three washouts of the hip have occurred. Whole body MRI (wbMRI) reveals a splenic abscess of 5 cm and a clinically undetected right shoulder septic arthritis and associated humeral osteomyelitis. Both collections are drained surgically.*

1. Your preferred **DIRECTED intravenous antibiotic** regimen in light of persistent S. aureus bacteraemia (once source control achieved) includes:  (select ALL antibiotics you would prescribe in your directed regimen for this patient)

Answers: flucloxacillin, cefazolin/cephalothin, vancomycin, clindamycin/lincomycin, cotrimoxazole, linezolid, rifampicin, ceftaroline, daptomycin, gentamicin, rifampicin, fusidate, other (please specify)

***Scenario B:****By day 8, blood cultures remain positive for*S. aureus*with vancomycin MIC of 2.0mg/L. Patient remains intubated and ventilated. TTE does not show evidence of endocarditis. Three washouts of the hip have occurred. Whole body MRI reveals a splenic abscess of 5 cm and a clinically undetected right shoulder septic arthritis and associated humeral osteomyelitis. Both collections are drained surgically.*

1. Your preferred **DIRECTED intravenous antibiotic** regimen in light of persistent S. aureus bacteraemia (once source control achieved) with rising vancomycin MIC includes:  (select ALL antibiotics you would prescribe in your directed regimen for this patient)

Answers: flucloxacillin, cefazolin/cephalothin, vancomycin, clindamycin/lincomycin, cotrimoxazole, linezolid, rifampicin, ceftaroline, daptomycin, gentamicin, rifampicin, fusidic acid, ceftobiprole, other (please specify)

*By day 10, blood cultures are negative*.

1. Your preferred total duration of **intravenous therapy** for this patient is:

Answers: 2 weeks, 4 weeks, 6 weeks, 8 weeks, 10 weeks, 12 weeks, other (please specify)

1. What influences you to treat for longer using **intravenous** therapy?

Answers: Persistent bacteraemia, persistent fevers, undrained focus, multifocal disease, anticipated poor adherence to oral regimen, other (please specify)

1. Would you consider **oral** step down therapy for this patient?

Answers: Yes, no, unknown

1. Your preferred methods to guide completion of therapy is (select all that apply)

Answers: Clinical assessment (if selected, please also list factors you use in 'Other' comments), CRP, ESR, imaging, FBC, other (please specify)

#### Any additional comments about the management of this case that you would like to make: Answers: free text

**Section four**

1. What do you think are important questions to address with clinical trials in the treatment of S aureus disease in children and adolescents?

Answers: free text

1. Would you enrol your patients with severe *S. aureus* infection [defined as any of ICU admission, multifocal disease, persistent bacteraemia > 5days, pneumonia, endocarditis, infant] in a trial of standard empiric therapy [as recommended in Therapeutic Guidelines: (flucloxacillin and/or vancomycin)] + placebo versus standard therapy + clindamycin?

Answers: yes, no

1. Please explain your response to the question above

Answer: free text

1. Could you please rank in order of priority [1 being your highest priority, 7 being your lowest priority] these evidence gaps in the treatment of *S. aureus* infection that need to be addressed? Please rank all items:

Answers: Adjunctive therapy for severe SA infection e.g clindamycin, duration of therapy for SA bacteraemia in children, step down antibiotics for SA osteomyelitis in children, use of whole body MRI in diagnostic workup of severe SA infection in children, paediatric and neonatal ICU management of severe SA infection, Camera 2 trial for children (flucloxacillin and vancomycin versus vancomycin alone for MRSA bacteraemia), new SA antimicrobials for children, optimal step down therapy for complicated MRSA infections in children, other (please specify)

1. Please complete this question if you included 'other' in your ranking for question 4 above (please specify)

**Thank you for your time in completing this survey!**
